# Prevalence of head and face actinic keratosis among older community residents aged ≥60 years in Shanghai: a cross-sectional study

**DOI:** 10.3389/fmed.2025.1558683

**Published:** 2025-08-11

**Authors:** Dekun Song, Yang Li, Lunhui Chang, Kai Liu, Yunwei Yang, Zhenlin Li, Ruiping Wang, Guolong Zhang

**Affiliations:** ^1^Institute of Photomedicine, Shanghai Skin Disease Hospital, Tongji University School of Medicine, Shanghai, China; ^2^Shanghai Huangpu District Yuyuan Community Health Service Center, Shanghai, China; ^3^The First Hospital of Anhui University of Science and Technology, Huainan, China; ^4^Shanghai Huangpu District Geriatric Nursing Hospital, Shanghai, China; ^5^Clinical Research and Innovation Transformation Center, Shanghai Skin Diseases Hospital, Medical School, Tongji University, Shanghai, China

**Keywords:** actinic keratosis, elderly population, dermoscopy, community, skin cancer, epidemiology, prevalence

## Abstract

**Background:**

Actinic keratosis (AK), as a key indicator of skin cancer risk, is vital for understanding the skin health status of the elderly population. However, the epidemiological data on the prevalence of AK among community residents in Shanghai remain limited. This study aimed to investigate the prevalence of AK in older Shanghai residents and explore the associated factors.

**Methods:**

A convenience sampling method was utilized to conduct a questionnaire survey among residents aged 60 years and above in a sub-district in Shanghai from March to June 2024. Data on participants’ demographic features, medical history, lifestyle, and skin conditions were collected, and a dermoscopic examination was performed on suspicious head and facial lesions to diagnose AK. In this study, a logistic regression model was applied to explore factors associated with the development of AK, and a *p* value less than 0.05 was considered statistically significant.

**Results:**

A total of 1,352 residents aged ≥ 60 years were recruited in the study, with 579 males (42.8%) and 773 females (57.2%). 79 AK cases were identified by dermoscopic examination, resulting in a prevalence rate of 5.84%. Residents diagnosed with AK in this study exhibited the following characteristics: older age, male predominance, higher rates of outdoor occupation and immunosuppressive drug use, increased prevalence of dry or mixed skin types, and lower skin health awareness. Logistic regression analysis indicated that older age was a risk factor for AK, with an odds ratio (OR) of 4.37 [95% confidence interval (CI): 1.02–18.83]. However, indoor work and good skin health knowledge were protective factors for AK, with the OR of 0.43 (95% CI: 0.24–0.77) and 0.57 (95% CI: 0.35–0.92), respectively.

**Conclusion:**

Age and outdoor work are significant risk factors for AK, while a higher level of skin health knowledge was protective against AK among older residents. The findings highlight the importance of early disease screening and skin health education among community residents.

## Introduction

Cutaneous squamous cell carcinoma (cSCC) is the second most common type of skin cancer, originating from the keratinocytes of the epidermis. Actinic keratosis (AK) and cSCC represent distinct stages within the same cancer progression spectrum, driven by key mutations such as p53, NOTCH, CDKN2A and MAPK (1, 2). AK is a precancerous lesion caused by chronic sun exposure, primarily occurring in fair-skinned individuals (Fitzpatrick Types I–II) of older age. This demographic prevalence explains why there are few reports on the epidemiology of the disease in other Fitzpatrick skin types, such as the Asian population (3). A recent meta-analysis estimated the global point prevalence of AK at 14% (4); however, this figure likely overestimates the prevalence in Asian populations, where studies report significantly lower rates (5–9) (e.g., 0.75–5% in Japanese populations, 0.51–7.1% in Taiwanese cohorts, and 2.7% in our previous study). Diagnosis of AK mainly relies on its clinical presentation, typically characterized by discrete, rough, poorly demarcated, skin-colored to lightly erythematous papules or plaques on sun-exposed regions, particularly the head and face (10). Consequently, most prior research on AK has concentrated on lesions in the head and facial areas (11). Dermoscopy offers a non-invasive and efficient method for AK diagnosis, demonstrating reported sensitivity and specificity rates of 98.7 and 95.0%, respectively (12).

The treatment of AK primarily involves local lesion removal through surgical interventions, physical/chemical modalities (e.g., cryotherapy, photodynamic therapy, chemical peeling, laser therapy), and pharmacologic agents (such as imiquimod, 5-fluorouracil, among others) (13). Most cases of AK have favorable prognoses; however, early intervention is critical due to the potential progression to cSCC (10). Amidst the escalating burden of disease in the aging global population, skin health is often overlooked, leading to a significant number of patients being diagnosed with advanced cSCC at their initial clinical presentation. In the United States, the direct medical costs attributed to AK patients amount to $1.2 billion annually, with indirect costs reaching $295 million (14). Although the data is concerning, it is also important to note that the potential for AK to progress to invasive cSCC is limited (15). Early photoprotection (e.g., UV avoidance, sunscreen) may reduce the development of new AK lesions, while therapeutic interventions (e.g., cryotherapy, topical agents) can prevent the progression of existing ones. Transitioning preventive measures from tertiary to secondary or even primary levels is essential for reducing healthcare costs while enhancing prognosis and quality of life outcomes (16). As a key indicator for assessing skin cancer risk (1, 17–20), AK is critical for understanding cutaneous health in older adults and guiding evidence-based public health strategies. This study investigates the prevalence, characteristics, and associated factors of AK on the head and face among older residents in a sub-district in Shanghai, China, to identify risk factors and provide guidance for the early prevention and treatment of AK.

## Methods

### Study population

The reporting of this study adheres to the STROBE guidelines. This study employed a comprehensive convenience sampling method to recruit residents aged 60 years and above in a sub-district in Shanghai between March and June 2024. This age threshold aligns with the standard definition of the elderly population in China and prior epidemiological studies. The selected community is a mature urban settlement with characteristic aging demographics and well-archived health records, making it suitable for an exploratory prevalence study. As a comprehensive screening initiative, we prioritized maximal coverage over predetermined sampling targets. Therefore, the final sample size (*N* = 1,352) was determined by the number of participants enrolled during the study period. This sample size is considered robust for the descriptive and exploratory aims of this study.

The inclusion criteria were as follows: (1) aged ≥60 years, both male and female; (2) permanent community residents in the Yuyuan sub-district; (3) understanding and willingness to participate in the questionnaire survey and dermoscopy examination. Non-inclusion criteria included: (1) individuals whose physical condition precluded them from dermoscopy examination and (2) residents with mental disorders and those who could not provide informed consent forms. This study was reviewed and authorized by the Institution Review Board of Shanghai Skin Diseases Hospital (2023–14), and informed consent was signed by each participant before the questionnaire interview. This study was conducted following the Declaration of Helsinki.

### Data collection

In this study, data of community residents were collected through face-to-face questionnaire interviews. The questionnaire consists of 4 parts: (1) demographic characteristics (gender, age, occupation, and educational attainment); (2) medical history (chronic diseases, neoplasm history, medication regimens, surgical interventions, allergic reactions, and familial medical backgrounds); (3) lifestyle habits (tobacco smoking and alcohol consumption, dietary patterns, exposure to chemical substances and physical radiation, ultraviolet radiation exposure, and sun protection practices); and (4) skin characteristics (Fitzpatrick skin typing, Glogau photoaging scale, periocular skin aging assessment, and sebum secretion classification).

### Dermoscopic examination

In this study, all participants were fully exposed to natural light to reveal their head and facial skin, and their skin conditions were meticulously documented. Standardized frontal and lateral photographs of the head and face were captured for archival purposes, and those with any suspicious skin lesions would undergo further dermoscopic examination. The duration of the comprehensive screening for each participant was approximately 20 min. This process, conducted by two experienced dermatologists, included standardized facial photographic archiving, visual lesion identification, and dermoscopic examination/documentation of all suspicious lesions. Standard handheld dermoscopes (FotoFinder bodystudio ATBM) were used.

### Definition and index calculation

In this study, age of older residents were classified into groups of <65 years, 65–70 years, 71–75 years and over 75 years. The type of occupation was documented as indoor work, outdoor work and both indoor and outdoor work. Sunlight exposure time (self-reported average daily cumulative duration outdoors during daylight hours, covering all activities) was categorized as less than 0.5 h, 0.5 to 1 h, 1 to 2 h, and over 2 h. Skin type was classified by trained dermatologists through standardized T-/U-zone sebum assessment and a Baumann-based questionnaire, synthesizing clinical and self-reported data into dry/normal/oily/mixed categories. Additionally, awareness of skin health and cancer prevention was evaluated using a 5-item questionnaire: (1) frequency of sun protection measures, (2) frequency of checking skin for new lesions, (3) recognition of risk factors (e.g., UV exposure), (4) ability to identify precancerous lesions through visual criteria, and (5) healthcare-seeking behavior for suspicious skin changes. Each item was scored on a 5-point Likert scale (e.g., 1 = “Never” to 5 = “Always”), yielding a total score range of 5–25. Based on pilot study data (n = 50), participants scoring above the median (12 points) were categorized as having adequate skin cancer prevention awareness.

### Quality control

In this study, each participant was selected following standard procedures. Two dermatologists jointly conducted all questionnaire surveys and dermoscopic examinations during a single clinical session, with immediate diagnostic consensus. Investigators received uniform training to ensure a comprehensive and consistent understanding of each item’s objective, significance, and completion requirements in the questionnaire survey and dermoscopic examination. All participants were investigated at the community health service centers, where they completed the questionnaires with ample assistance from the investigators. The questionnaires were reviewed on-site to ensure the completeness of the information. All data were collected through on-site questionnaire surveys and dermatological examinations. The data entry process involved double-checking to ensure high data quality. Most lesions could be diagnosed clinically using dermoscopic examination, while pathological diagnosis was conducted for those that could not be clarified.

### Statistical analysis

In this study, data analysis was performed using SPSS 27.0 statistical software. The analyses were performed on a complete-case basis, with participants excluded if they had missing data on key variables. Quantitative data are mean and standard deviation (SD) or median and interquartile range (IQR) as appropriate. Student’s t-test and Mann–Whitney U test were applied to correspondingly assess the difference between groups. Frequency count and proportion (percentage) were calculated for qualitative variables, and the *χ*^2^ test was used to analyze the difference between groups. Odds ratios (OR) and 95% confidence intervals (CI) were calculated by univariate and multivariate logistic regression analysis to explore the association between various exposure factors and the risk of AK. In this study, a *p* value less than 0.05 (two-tailed) was considered statistically significant.

## Results

**Figure 1:**
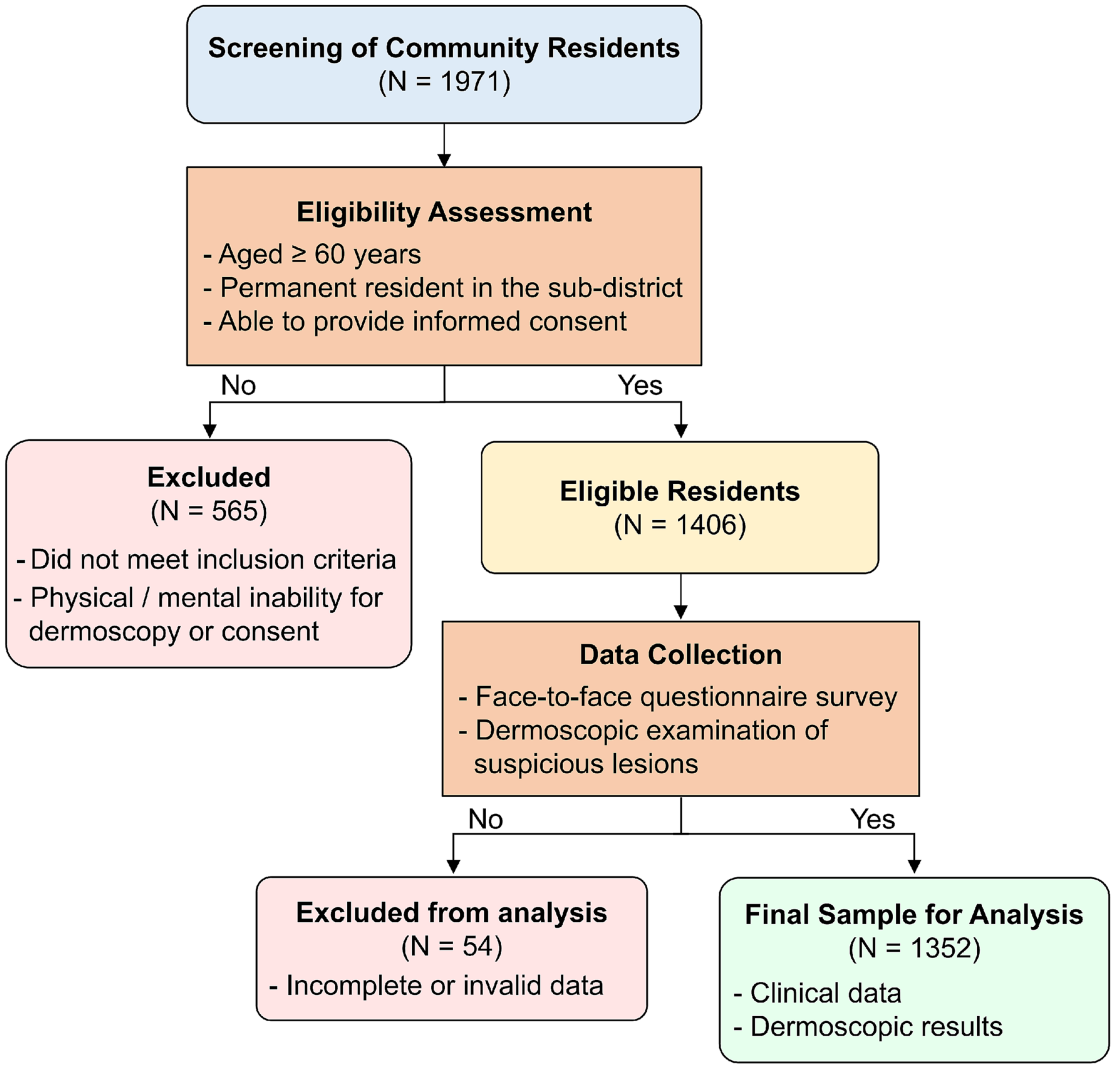
Flowchart of participant recruitment and enrollment process.

From an initial community cohort of 1,971 residents, 1,406 eligible participants completed questionnaires. Of these, 1,352 valid responses were included in the final analysis, as detailed in Figure 1. Most participants aged between 65 and 75 years, with an average age of (71.2 ± 5.2) years. Among them were 579 males (42.8%) and 773 females (57.2%). In terms of work types, 1,043 individuals (77.1%) were indoor workers, 143 (10.6%) were outdoor workers, and 166 (12.3%) had both indoor and outdoor work.

In this study, the average ages were (72.4 ± 5.3) years for males and (71.2 ± 5.0) years for females. The proportion of females engaged in indoor work is significantly higher than that of males (85.3% vs. 66.3%). Males had a higher prevalence of type 2 diabetes mellitus, hypertension, heart diseases, and neurological disorders than females. Regarding lifestyle habits, males had a significantly higher prevalence of alcohol consumption, tobacco smoking and chemical hazards exposure. Additionally, males had significantly longer sun exposure times than females and a higher proportion with a history of sunburn (9.2% vs. 5.6%). In terms of skin conditions, males had a higher proportion of oily skin (55.1% vs. 39.6%) and mixed skin (20.6% vs. 16.8%) (Table 1).

**Table 1:** Demographic features of people aged ≥60 years in Huangpu District, Shanghai, China (*n* = 1,352).

Variables	All (*n* = 1,352)	Male (*n* = 579)	Female (*n* = 773)	*t*/*χ*^2^	*p*
Age (years), mean (SD)	71.2 (5.2)	72.4 (5.3)	71.2 (5.0)	4.44	<0.01
Age (years), *n* (%)				16.3	<0.01
<65	100 (7.4)	36 (6.2)	64 (8.3)		
65–70	543 (40.2)	207 (35.8)	336 (43.5)		
71–75	353 (26.1)	155 (26.8)	198 (25.6)		
>75	356 (26.3)	181 (31.3)	175 (22.6)		
Type of occupation, *n* (%)				67.24	<0.01
Indoor work	1,043 (77.1)	384 (66.3)	659 (85.3)		
Outdoor work	143 (10.6)	90 (15.5)	53 (6.9)		
Both indoor and outdoor	166 (12.3)	105 (18.3)	61 (7.9)		
Noncommunicable diseases, *n* (%)					
Type 2 diabetic mellitus	301 (22.3)	144 (24.9)	157 (20.3)	3.97	<0.05
Hypertension	729 (53.9)	334 (57.7)	395 (51.1)	5.78	0.02
Hyperlipidemia	410 (30.3)	130 (22.5)	280 (36.2)	29.69	<0.01
Heart diseases	142 (10.5)	74 (12.8)	68 (8.8)	5.59	0.02
Liver diseases	23 (1.7)	6 (1.0)	17 (2.2)	2.68	0.11
lung tuberculosis	13 (1.0)	4 (0.7)	9 (1.2)	0.78	0.38
Neurological disorders	34 (2.5)	22 (3.8)	12 (1.6)	6.81	<0.01
Photosensitive drugs history, *n* (%)	802 (59.3)	353 (60.9)	449 (58.1)	1.14	0.29
Rheumatic immunity disease history, *n* (%)	30 (2.2)	5 (0.9)	25 (3.2)	8.57	<0.01
Immunosuppressive drugs history, *n* (%)	14 (1.0)	1 (0.2)	13 (1.7)	7.35	<0.01
Cancer history, *n* (%)	89 (6.6)	30 (5.2)	59 (7.6)	3.23	0.07
Allergy history, *n* (%)	186 (13.8)	62 (10.7)	124 (16.0)	7.93	<0.01
Alcohol drinking, *n* (%)	155 (11.5)	138 (23.8)	17 (2.2)	152.55	<0.01
Tobacco smoking, *n* (%)	241 (17.8)	235 (40.6)	6 (0.8)	357.92	<0.01
Chemical exposure history, *n* (%)	344 (25.4)	164 (28.3)	180 (23.3)	4.43	0.04
Photosensitive vegetables, *n* (%)	1,104 (81.7)	470 (81.2)	634 (82.0)	0.16	0.69
Photosensitive fruits, *n* (%)	874 (64.6)	376 (64.9)	498 (64.4)	0.04	0.85
Photosensitive seafood, *n* (%)	571 (42.3)	252 (43.5)	319 (41.3)	0.69	0.41
Time of sunlight exposure, *n* (%)				28.1	<0.01
Less than 0.5 h	399 (29.5)	128 (22.1)	271 (35.1)		
0.5–1 h	418 (30.9)	196 (33.9)	222 (28.7)		
1–2 h	349 (25.8)	160 (27.6)	189 (24.5)		
Over 2 h	186 (13.8)	95 (16.4)	91 (11.8)		
Sunscreen measures, *n* (%)	452 (33.4)	201 (34.7)	251 (32.5)	0.75	0.39
History of sunburn, *n* (%)	96 (7.1)	53 (9.2)	43 (5.6)	6.47	0.01
Knowledge of skin cancer, *n* (%)	798 (59.0)	336 (58.0)	462 (59.8)	0.42	0.52
Skin type, *n* (%)				119.03	<0.01
Dry skin	135 (10.0)	79 (13.6)	56 (7.2)		
Normal skin	343 (25.4)	62 (10.7)	281 (36.4)		
Oily skin	625 (46.2)	319 (55.1)	306 (39.6)		
Mixed skin	249 (18.4)	119 (20.6)	130 (16.8)		

Not all metrics are depicted in the tables due to space limitations. SD, Standard deviation.

### Comparison of characteristics between AK and non-AK groups

In this study, 79 out of 1,352 surveyed residents were diagnosed with AK; the prevalence of AK among the older residents was 5.8%. Most cases were confirmed on-site via dermoscopy, while 8 initially inconclusive cases were subsequently diagnosed through histopathological examination. Moreover, we have also identified 5 cases of basal cell carcinoma (BCC) and 2 cases of cSCC, with a prevalence of 0.37 and 0.15%, respectively. Residents with AK had a significantly higher average age (74.8 ± 5.9 years) compared to those without AK (71.5 ± 5.1 years). The proportion of males, outdoor workers, and individuals using immunosuppressive drugs was all higher among residents with AK than those without AK. For different skin types, residents with AK had a higher proportion of dry and mixed skin types than those without AK. Meanwhile, residents with AK had significantly lower awareness of skin health and cancer prevention (Table 2).

**Table 2:** The prevalence of AK among people aged ≥60 years old in Huangpu District, Shanghai, China (*n* = 1,352).

Variables	All (*n* = 1,352)	AK (*n* = 79)	None-AK (*n* = 1,273)	*t*/*χ*^2^	*p*
Age (years), mean (SD)	71.2 (5.2)	74.8 (5.9)	71.5 (5.1)		<0.01
Gender *n* (%)				5.67	0.02
Male	579 (42.8)	44 (7.6)	535 (92.4)		
Female	773 (57.2)	35 (4.5)	738 (95.5)		
Type of occupation, *n* (%)				16.29	<0.01
Indoor work	1,043 (77.1)	53 (5.1)	990 (94.9)		
Outdoor work	143 (10.6)	19 (13.3)	124 (86.7)		
Both indoor and outdoor	166 (12.3)	7 (4.2)	159 (95.8)		
Noncommunicable diseases, *n* (%)				0.31	0.58
Yes	1,062 (78.6)	64 (6.0)	998 (94.0)		
No	290 (21.4)	15 (5.2)	275 (94.8)		
Photosensitive drugs history, *n* (%)	802 (59.3)	50 (63.3)	752 (59.1)	0.55	0.46
Rheumatic immunity disease history, *n* (%)	30 (2.2)	4 (5.1)	26 (2.0)	3.13	0.07
Immunosuppressive drugs history, *n* (%)	14 (1.0)	3 (3.8)	11 (0.86)	6.24	0.02
Cancer history, *n* (%)	89 (6.6)	8 (10.1)	81 (6.4)	1.71	0.19
Allergy history, *n* (%)	186 (13.8)	10 (12.7)	176 (13.8)	0.09	0.77
Alcohol drinking, *n* (%)	155 (11.5)	5 (6.3)	150 (11.8)	2.18	0.14
Tobacco smoking, *n* (%)	241 (17.8)	18 (22.8)	223 (17.5)	1.41	0.24
Chemical exposure history, *n* (%)	344 (25.4)	22 (27.9)	322 (25.3)	0.26	0.61
Photosensitive vegetables, *n* (%)	1,104 (81.7)	66 (83.5)	1,038 (81.5)	0.20	0.66
Photosensitive fruits, *n* (%)	874 (64.6)	50 (63.3)	824 (64.7)	0.07	0.80
Photosensitive seafood, *n* (%)	571 (42.3)	39 (49.4)	532 (41.8)	1.75	0.19
Time of sunlight exposure, *n* (%)				3.17	0.37
Less than 0.5 h	399 (29.5)	22 (5.5)	377 (94.5)		
0.5–1 h	418 (30.9)	21 (5.0)	397 (94.9)		
1–2 h	349 (25.8)	20 (5.7)	329 (94.3)		
Over 2 h	186 (13.8)	16 (8.6)	170 (91.4)		
Sunscreen measures, *n* (%)	452 (33.4)	25 (31.6)	427 (33.5)	0.12	0.73
History of sunburn, *n* (%)	96 (7.1)	7 (8.9)	89 (7.0)	0.39	0.53
Knowledge of skin cancer, *n* (%)	798 (59.0)	33 (41.8)	765 (60.1)	10.32	<0.01
Skin type, *n* (%)				9.37	0.02
Dry skin	135 (10.0)	11 (8.2)	124 (91.9)		
Normal skin	343 (25.4)	18 (5.3)	325 (94.8)		
Oily skin	625 (46.2)	27 (4.3)	598 (95.7)		
Mixed skin	249 (18.4)	23 (9.2)	226 (90.8)		

SD, Standard deviation.

### Factors associated with AK among older residents

In this study, the univariate logistic regression analysis indicated that residents with older age [(OR = 1.97, 95% CI 0.46–8.54) for 65–70 group; (OR = 3.10, 95% CI 0.71–13.45) for 71–75 group; (OR = 5.34, 95% CI 1.26–22.61) for >75 group] had a higher prevalence of AK. However, residents with female gender (OR = 0.58, 95% CI 0.37–0.91), with indoor work or indoor and outdoor work [(OR = 0.35, 95% CI 0.20–0.61) for indoor work; (OR = 0.29, 95% CI 0.12–0.71) for indoor and outdoor work], with good knowledge of skin cancer (OR = 0.48, 95% CI 0.30–0.76) had a lower prevalence of AK. For skin types, residents with dry skin (OR = 1.60, 95% CI 0.74–3.49) and with mixed skin (OR = 1.84, 95% CI:1.00–3.49) had higher AK prevalence, while those with oily skin had lower AK prevalence (OR 0.82, 95% CI 0.44–1.50; Table 3, Model A).

**Table 3:** The prevalence of AK and its associated influencing factors among people aged ≥60 years old in Huangpu district, Shanghai, China.

Variables	Prevalence of AK, *n* (%)	Model A		Model B		Model C	
		OR	95% CI	OR	95% CI	OR	95% CI
Age (years)ǂ							
<65	2 (2.0)	1.00	–	1.00	–	1.00	–
65–70	21 (3.9)	1.97	0.46–8.54	1.99	0.45–8.74	1.91	0.44–8.34
71–75	21 (6.0)	3.10	0.71–13.45	2.98	0.67–13.17	2.84	0.65–12.54
>75	35 (9.8)	***5.34***	***1.26–22.61***	***4.66***	***1.07–20.29***	***4.37***	***1.02–18.83***
Genderǂ							
Male	44 (7.6)	1.00	–	1.00	–	1.00	–
Female	35 (4.5)	***0.58***	***0.37–0.91***	0.70	0.40–1.24	0.66	0.40–1.08
Type of occupationǂ							
Outdoor work	19 (13.3)	1.00	–	1.00	–	1.00	–
Indoor work	53 (5.1)	***0.35***	***0.20–0.61***	***0.45***	***0.25–0.81***	***0.43***	***0.24–0.77***
Both indoor and outdoor	7 (4.2)	***0.29***	***0.12–0.71***	***0.34***	***0.14–0.87***	***0.33***	***0.13–0.82***
Knowledge of skin cancerǂ							
Yes	33 (4.1)	***0.48***	***0.30–0.76***	***0.55***	***0.33–0.90***	***0.57***	***0.35–0.92***
No	46 (8.3)	1.00	–	1.00	–	1.00	–
Skin type, *n* (%)ǂ							
Normal skin	18 (5.3)	1.00	–	1.00	–	1.00	–
Dry skin	11 (8.2)	1.60	0.74–3.49	1.18	0.52–2.68	1.18	0.52–2.66
Oily skin	27 (4.3)	0.82	0.44–1.50	0.71	0.37–1.35	0.72	0.37–1.36
Mixed skin	***23 (9.2)***	***1.84***	***1.00–3.49***	1.39	0.70–2.73	1.41	0.72–2.76
Time of sunlight exposure							
Less than 0.5 h	22 (5.5)	1.00	–	1.00	–		
0.5–1 h	21 (5.0)	0.91	0.49–1.68	0.98	0.52–1.84		
1–2 h	20 (5.7)	1.04	0.56–1.94	1.07	0.56–2.03		
Over 2 h	16 (8.6)	1.62	0.83–3.15	1.36	0.67–2.75		
Noncommunicable diseases							
Yes	64 (6.0)	1.18	0.66–2.10	1.17	0.64–2.12		
No	15 (5.2)	1.00	–	1.00	–		
Tobacco smoking							
Yes	18 (7.5)	1.39	0.81–2.40	1.11	0.58–2.13		
No	61 (5.5)	1.00	–	1.00	–		
Sunscreen measures							
Yes	25 (5.5)	0.92	0.56–1.50	1.20	0.72–2.03		
No	54 (6.0)	1.00		1.00	–		

ǂThe differences between groups were statistically significant (*p* < 0.05). These values are presented in bold. Model A: Uni-variate logistic regression to explore factors associated with the prevalence of AK among people aged ≥60 years in Shanghai. Model B: Multivariate logistic regression to explore factors associated with the prevalence of AK among people aged ≥60 years with the mutual adjustment of age, gender, occupation, skin cancer knowledge, skin type, sunlight exposure times, non-communicable diseases, tobacco smoking and sunscreen measure. Model C: Multivariate logistic regression to explore factors associated with the prevalence of AK among people aged ≥60 years with the adjustment of age, gender, occupation, skin cancer knowledge and skin type. OR, Odds ratio; CI, Confidence interval.

In this study, we established two multivariate logistic regression models to explore factors associated with AK among older community residents. In model B, which included all independent variables, the results showed that older community residents had a higher prevalence of AK [(OR = 1.99, 95% CI 0.45–8.74) for 65–70 group, (OR = 2.98, 95% CI 0.67–13.17) for 71–75 group, and (OR = 4.66, 95% CI: 1.07–20.29) for >75 group]. However, residents who work indoors or indoors and outdoors [(OR = 0.45, 95% CI 0.25–0.81) for indoor work; (OR = 0.34, 95% CI 0.14–0.87) for indoor and outdoor work], with good knowledge of skin cancer (OR = 0.55, 95% CI 0.33–0.90) had a lower prevalence of AK. In Model C, only variables showing statistical significance (*p* < 0.05) in univariate logistic regression were retained to construct a parsimonious multivariate model. This approach is often preferable for studies with a moderately sized sample, as it helps avoid overfitting and enhances interpretability. The results also indicated that older residents had a higher prevalence of AK [(OR = 1.91, 95% CI 0.44–8.34) for the 65–70 group, (OR = 2.84, 95% CI 0.65–12.54) for the 71–75 group, and (OR = 4.37, 95% CI 1.02–18.83) for the >75 group]. Whereas residents who work indoors or indoors and outdoors [(OR = 0.43, 95% CI 0.24–0.77) for indoor work; (OR = 0.33, 95% CI 0.13–0.82) for indoor and outdoor work] or with good knowledge of skin cancer (OR = 0.57, 95% CI 0.35–0.92) had a lower prevalence of AK (see Table 3). Furthermore, sensitivity analysis employing a liberal inclusion threshold (univariate *p*-value < 0.20) demonstrated that the additionally incorporated variable remained statistically non-significant, and the odds ratios of the core predictors identified in Model C were not materially altered (changed by less than 5%), which supported the robustness of our final model.

## Discussion

To our knowledge, this study is the first large-sample investigation to explore the AK prevalence and associated risk factors among older community residents in China. The findings indicate that the prevalence of AK was 5.84%, which was relatively lower than that reported in Western countries. Moreover, age and outdoor work are significant risk factors for AK, while a higher level of skin health knowledge was a protective factor against AK among older residents.

Epidemiological studies on AK in Asian populations are relatively lacking, with limited reports indicating that the incidence rate of AK in older adults is between 1.05 and 10.59 per 10,000 person-years (21–23). Few studies have addressed the prevalence of AK in Asia in recent years. In this study, we report that the prevalence rate of AK on the head and face among older residents in Shanghai has reached 5.8%, whereas our team’s previous epidemiological study conducted in 2011 showed a prevalence of 2.7% for AK on the head and face (9). The prevalence of AK among the older residents may have significantly increased, a phenomenon that corresponds with the results of other studies with substantial time spans (24). Apart from the impact of intensified population aging, this may be associated with environmental and lifestyle changes. UV exposure is the most significant risk factor for the development of AK. In recent years, the continuous decline in the thickness of the atmospheric ozone layer, the burning of chemical fuels, and global warming have led to increasing exposure to UV radiation (25). In terms of lifestyle, the significant improvement in transportation convenience has made travel to sunny vacation destinations a popular choice for older adults, which also leads to increased exposure to UV radiation (26).

In addition to the actual increase in AK cases, the rise in screening rates and the improvements in diagnostic technology may contribute to the observed increase in prevalence, as this has effectively identified cases that may have previously escaped diagnosis (27). While a prior Chinese hospital-based study reported a lower AK prevalence (1.05 per 1,000 in the cross-sectional study) among general dermatology patients (28)–likely underestimated (21)–our substantially higher prevalence stems from fundamental methodological distinctions. Specifically, our study targeted a high-risk population and implemented proactive screening protocols combined with systematic dermoscopic evaluation of all suspicious lesions, thereby enhancing the detection of subclinical AK. This underscores the importance of active AK screening in clinical practice. Dermoscopy is a non-invasive diagnostic technique that, while being quick and accessible, also ensures good sensitivity and specificity in detecting various skin diseases and is now widely used in large public hospitals in China (29). It not only allows for the diagnosis of AK but also enables the stratification of AK into three grades, which is very helpful for guiding clinical treatment (30).

Age is the predominant risk factor for AK, consistent with previous studies (25, 31, 32). Aging is characterized by compromised clearance of aberrant cells and the buildup of detrimental factors, which collectively facilitate cellular oncogenic transformation (33). Previous research has found that age-related alterations in the dermal microenvironment play a pivotal role in tumor initiation, with fibroblasts promoting the uncontrolled proliferation of precancerous keratinocytes through the secretion of cytokines and other mediators (34). Gender also impacts AK prevalence, with women typically having lower rates due to less outdoor activity and greater sun protection. The fact that gender is not a significant factor in multivariate analysis further indicates that the differences in AK prevalence are attributable to gender-associated external factors rather than inherent differences in susceptibility.

A history of outdoor work was identified as an important risk factor for AK. Since most older individuals have been retired for many years, this persistent association may come from the post-effects of light exposure (35). This is consistent with previous studies reporting a positive correlation between sunburn history and skin cancer (36, 37). UV irradiation of the skin leads to the formation of DNA photoproducts such as cyclobutane pyrimidine dimers (CPDs) and pyrimidine-pyrimidone (6–4) photoproduct (6–4PP) (38). If unrepaired, these damages can alter keratinocyte DNA, leading to oncogene activation and tumor suppressor gene inactivation, with TP53 mutations—the most common tumor suppressor defect in skin cancer—being a key driver (39). Subsequently, various mechanisms promoting cell proliferation are activated, driving the onset and progression of AK and cSCC. Additionally, UV can induce immunosuppression, playing an indirect promotional role in this process (40). These changes exhibit a cumulative effect, persisting over many years following the initial damage (41). Our *in vivo* SKH-1 mouse experiments validate the hypothesis; despite terminating UV irradiation (Solar UV Simulator, SIGMA, Shanghai, China) in the absence of overt skin phenotypes, a significant proportion of mice subsequently developed AK and even cSCC over months, suggesting sustained carcinogenic effects of ultraviolet exposure (42).

In a previous study, we observed no significant impact of the overall level of education on the prevalence of AK. Therefore, this survey incorporated specific items to reflect skin protection and cancer prevention knowledge levels. Our analysis revealed that a better understanding of skin health is instrumental in reducing the incidence of AK, highlighting the paucity of skin health knowledge within the current general education curriculum in our country (35). Evidence-based strategies, which include distributing educational brochures and delivering structured mini-lectures, have effectively enhanced health knowledge dissemination among target populations (43, 44). High-incidence countries like the United States and Australia have already established more comprehensive guidelines for prevention measures. Specifically, these measures include behavioral counseling, regular self-skin assessment, and the enhancement of sun protection measures (including the use of high SPF sunscreens, physical barriers, avoidance of intense sun exposure, and refraining from the use of indoor tanning beds) (12, 45–47). Despite the low positive correlation between UV exposure and keratinocyte carcinoma incidence in East Asian populations (48), adequate UV protection remains a safe and effective option. For patients diagnosed with AK, sun protection measures are strongly recommended by the guidelines (17). This underscores the importance of continued education and preventive measures in managing and reducing the prevalence of AK and cSCC.

We initially included Fitzpatrick skin typing and some other indicators. However, we observed minimal heterogeneity within our surveyed population—predominantly Asians with Fitzpatrick skin types III or IV—and no significant statistical correlation with AK, aligning with prior surveys. Therefore, we adopted the more commonly used skin sebum secretion classification for daily skin assessment in Asians, categorizing skin as normal, dry, oily, or mixed (oily T-zone with relatively dry cheeks) for statistical analysis (49). We found that individuals with dry or mixed skin types demonstrated a higher prevalence of AK. While mechanistic understanding remains incomplete, reduced sebum secretion—particularly on photo-exposed malar cheeks, a high-risk AK site—may compromise epidermal barrier integrity. Sebum physically attenuates UV penetration and contains photoprotective constituents (e.g., squalene, vitamin E) that mitigate oxidative damage (50–53). However, its direct role in AK pathogenesis requires further investigation. Additionally, immunosuppressive medications may have a significant impact on the development of AK, although the limited amount of data, to some extent, restricts subsequent practical analysis. However, in our actual survey process, we did find that patients on such medications developed AK at a younger age and with more severe conditions, consistent with previous studies (31, 54). Our study did not find a positive association between diet and AK prevalence, possibly due to insufficiently detailed food categorization and quantification of intake (55).

This study comes with inherent limitations. Firstly, as a cross-sectional study, it does not allow for establishing a causal link between exposure factors and the risk of AK on the head and face in the elderly population. Further validation requires the conduct of prospective cohort studies or randomized controlled trials. Secondly, due to the limited sample size, many exposure factors had too few positive cases, which precluded their inclusion in statistical analyses and tabular presentations, or the statistical results indicated no significant differences. Thirdly, lifestyle-related risk factors (e.g., sun exposure habits, smoking history) are particularly prone to recall bias due to retrospective self-reporting, which may affect exposure classification accuracy. Moreover, we relied on a convenience sampling from a single urban sub-district. This non-probabilistic sampling method introduces a potential for selection bias and limits the generalizability of our findings. Consequently, the prevalence of AK reported here may not be representative of the broader elderly population in Shanghai or other regions. Future large-scale, multi-center studies using probabilistic sampling are required to validate these findings. Lastly, the majority of AK diagnoses were based on clinical and dermoscopic criteria without histopathological confirmation, which may lead to a modest overestimation of prevalence.

In conclusion, the prevalence of AK on the head and face among older residents in the Shanghai area is high, associated with three main factors (age, outdoor work, and skin health awareness), and has shown an increasing trend over the past decade (from 2.7 to 5.8%). For older individuals, especially those with a higher risk of AK due to advanced age or a history of outdoor work, implementing appropriate preventive and screening measures, coupled with proactive skin health education, represents a cost-effective medical strategy (56).

## Data Availability

The raw data supporting the conclusions of this article will be made available by the authors upon reasonable request.
